# SEM analysis of agreement with regulating online hate speech: influences of victimization, social harm assessment, and regulatory effectiveness assessment

**DOI:** 10.3389/fpsyg.2023.1276568

**Published:** 2023-12-19

**Authors:** Ahran Park, Minjeong Kim, Ee-Sun Kim

**Affiliations:** ^1^School of Media and Communication, Korea University, Seoul, Republic of Korea; ^2^Division of Media and Communication, Hankuk University of Foreign Studies, Seoul, Republic of Korea; ^3^Department of Sociology, Seoul National University, Seoul, Republic of Korea

**Keywords:** online hate speech, experience of victimization, social harm assessment, regulation effectiveness, SEM analysis

## Abstract

In an era where digital interactions are increasingly prevalent, the challenge of effectively regulating online hate speech has emerged as a crucial societal concern. Balancing the regulation of such speech with the preservation of online freedom of expression is a delicate task, requiring broad consensus among internet users. This study delves into the various factors shaping public attitudes towards the regulation of online hate speech in South Korea. An online survey of 1,000 Internet users provided the data for a structural equation model. Our findings reveal that experiences of victimization by hate speech, online activity such as content uploading, assessment of social harm caused by online hate speech, and assessment on the effectiveness of regulatory measures all play significant roles in garnering support for regulation. Notably, online activity correlates strongly with increased encounters with hate speech. This, in turn, leads to a more profound understanding of its social harm and, consequently, a heightened inclination to support regulatory measures. These insights underscore the growing urgency to address online hate speech, especially as online activity continue to intensify. This study contributes to the discourse on online hate speech regulation by highlighting the complex interplay of personal experience, perceived harm, and efficacy of regulation in shaping public consensus.

## 1 Introduction

The persistence of online hate speech remains a pressing concern, exacerbated during the COVID-19 pandemic, where disinformation and misinformation further strained societies (Perez, 2021). Hate speech is a significant form of malicious behavior on digital platforms that necessitates regulatory interventions (Bennett, 2016). While European nations have been proactive in enacting legislation to curb hate speech, the United States has emphasized free speech principles, limiting regulations to speech that incites violence (Tsesis, 2009; Bleich, 2014).

Digital platforms’ algorithms have facilitated the rapid dissemination of hateful content, compounding this issue. Recent research has revealed a surge in hateful tweets following Twitter’s acquisition by Elon Musk in October 2022, which led to fewer content moderation restrictions (Hickey et al., 2023). The devastating consequences of hate speech extend from the psychological distress experienced by targeted individuals to real-world discrimination and violence. Furthermore, the spread of such hate messages fuels extremism and social polarization.

This study is set against the backdrop of South Korea, where hate speech targeting the LGBTQ+ community, women, and foreigners has become a prominent social issue since the 2010s (Choi et al., 2022). Korean criminal law does not explicitly address hate speech, often resulting in the use of defamation and insult laws to prosecute such cases. This gap has sparked legislative initiatives aimed at introducing anti-discrimination laws to specifically tackle hate speech. Nevertheless, the definition of hate speech and the extent of regulation remain contentious issues, stalling the adoption of such laws.

Despite these legislative hurdles, public sentiment in Korea strongly favors the regulation of hate speech. A survey by the Korea Press Foundation in 2016, involving 1,000 participants, revealed that an overwhelming 77.1% recognized the need for hate speech regulation (Korea Press Foundation, 2016). The support for this regulation is rooted in concerns that hate speech disrupts societal harmony (37.0%), perpetuates discrimination against marginalized groups (28.5%), and imparts negative values to the younger generation (24.0%). Yet there is a dearth of empirical studies investigating the motivations behind citizens’ advocacy for hate speech regulation.

Previous research has explored people’s willingness to impose restrictions on hate speech in different countries (Reddy, 2002; Kvam, 2015; Muddit, 2015). However, little research has been conducted on user experiences and perceptions of online hate speech and regulation (Shim, 2022). This study aims to address this gap by investigating the impact of online hate speech on users and identifying the factors that shape citizens’ attitudes and consensus regarding hate speech regulation, within the specific social context of South Korea. This study raises the following research questions. First, what are the factors that influence agreement with the regulation of online hate speech? Second, how does the experience of being attacked online affect the evaluation of the social harms of online hate speech and the attitudes toward its regulation? Third, how does online uploading activity affect the experience, evaluation, and attitudes toward regulation of online hate speech? The conclusion of the study posits that the support of citizens for online regulation is significantly influenced by two major factors: the impact of online hate speech and the perceived effectiveness of regulatory measures.

## 2 Theoretical model and research questions/hypotheses

### 2.1 The chilling effect of hate speech on freedom of expression

Freedom of expression holds a fundamental place in supporting personal autonomy and sustaining democracy. Numerous scholars have voiced concerns against regulating hate speech, emphasizing the need to preserve free speech rights. For instance, Robert Post, a distinguished First Amendment scholar, has argued that public discourse is crucial for forming a democratic collective will, leading him to suggest that racist speech should remain unregulated within the realm of public discourse (Tsesis, 2009). Consequently, the predominant stance in both jurisprudence and public debate regarding the regulation of hate speech has been to strike a balance between the freedom of speech and protecting the dignity and autonomy of those targeted by hate speech. One of the primary concerns in implementing mandatory measures to limit hate speech is the potential chilling effect such regulation might have on free expression. This concern underlines the complexity of navigating between upholding free speech and curtailing hate speech.

However, this approach considers only one side of the coin, the speaker’s right to free speech. Respecting listeners’ right to free speech is equally important. The following is an illustrative example. Charles Coughlin, an anti-Semite and pro-fascist in the late 1930s in the United States, gained national attention as a successful radio performer and a controversial and divisive figure (Goodman, 2015). His broadcasts were distressing to some listeners and celebrated by others, which inevitably sparked debates about freedom of speech. Goodman (2015) analyzed letters from Coughlin listeners to compare them with the policy history of free speech regulation. He found that pro-Coughlin listeners attempted to make a civil rights issue out of some stations’ refusal to sell time to Coughlin, while anti-Coughlin listeners documented the pain and harm caused by the broadcasts and argued that Coughlin’s right to broadcast must be limited to protect listeners’ rights (Goodman, 2015).

A similar sentiment emerged in a 2017 European survey. According to the survey findings, 75% of those who followed or participated in online debates had encountered instances of abuse, threats, or hate speech, and almost half of these respondents said that this deterred them from engaging in online discussions (European Commission, 2017). Meanwhile, Penney (2017) found that online harassment/cyberbullying laws, which criminalized online speech intending to “harass or intimidate another person” may lead to more speech and sharing online—especially from women, the often victims of these malicious activities online—while only minimally impacting other forms of speech and expression. To summarize, unregulated hate speech may have a chilling effect on the free speech of those targeted by it.

As these findings show, exploring the actual effects of regulations on people’s attitudes and behaviors through empirical studies is crucial. Normative legal debates and examining the societal consensus on the regulation of online hate speech are equally important. This study is designed to investigate the influence of online hate speech on users by exploring the factors that shape citizens’ consensus on hate speech regulation.

### 2.2 Agreement with the regulation: direct experience and social harm assessment

Previous research has identified various personality traits, attitudinal factors, and cultural influences as key determinants of individuals’ support for freedom of expression (Lambe, 2004; Downs and Cowan, 2012). A 2018 survey by the Knight Foundation found that American university students, especially women, African Americans, and Democrats, tend to prioritize diversity and inclusion on college campuses over the right to freedom of expression (Knight Foundation, 2018). However, there is a scarcity of research exploring how citizens’ perceptions of the social harm caused by online hate speech influence their attitudes towards its regulation.

This research hypothesizes a correlation between individuals’ exposure to online hate speech and their willingness to comply with regulations designed to curb such speech. In addition, it formulates a research question to delve into how this exposure affects individual compliance with regulatory frameworks governing online hate speech. The hypothesis is centered around the idea that the perceived social harm of online hate speech plays a critical role in shaping individuals’ support for regulations. Accordingly, the research question posited is:

R1. Does the perceived social harm of online hate speech affect individuals’ level of agreement with regulations aimed at its control?

This study aims to explore the relationship between users’ exposure to online hate speech and their agreement with regulatory measures. As online hate speech becomes more prevalent, internet users are increasingly encountering it, which could lead to two contrasting effects on their views regarding regulation. This hinges on the nature of their exposure. For individuals who often come across hate speech but are not directly targeted, there might be a desensitization effect. They might start viewing hate speech as less alarming, leading to a preference for less stringent regulation. This is in line with the desensitization effect theory (Cline et al., 1973; Thomas et al., 1977; Linz et al., 1984, 1988, 1989; Krafka et al., 1997; Funk et al., 2004), which suggests that increased exposure to harmful content can lead to desensitization. Desensitization involves a diminished recognition of hate speech as a serious issue, essentially underplaying its negative societal impact and normalizing it within daily life.

Conversely, individuals who not only encounter hate speech more frequently but also become its victims may develop a heightened awareness of its damaging effects. This awareness could foster a stronger demand for regulatory measures. Although existing research does not provide substantial evidence for this, the study introduces the following research question:

R2. Does frequent victimization by online hate speech influence the level of agreement with the regulation of hate speech online?

When considering the regulation of online hate speech, the third-person effect is a crucial factor. Originating from Davison’s (1983) work in this effect suggests that people often believe mass media messages have a greater impact on others than on themselves. While initially focused on traditional mass media, the concept has since been expanded to include digital media (Kim and Hancock, 2015; Lev-On, 2017). For example, Buturoiu et al. (2017) found that Facebook users who deemed content undesirable tended to assess its impact as more significant on others than on themselves. Similarly, Guo and Johnson (2020) observed that university students often perceive hate speech as more impactful on others, which correspondingly influenced their support for censorship of such speech and their readiness to report hateful content.

Regarding online hate speech, we expect that the third-person effect is not an isolated phenomenon, but functions in conjunction with user experience. Based on this premise, the following hypothesis is proposed:

*H1*. Users’ victimization experiences with online hate speech influence their regulatory views, and this influence is mediated by an assessment of the social harm of online hate speech.

### 2.3 Agreement with the regulation: online activity and regulatory effectiveness assessment

Research has established a negative correlation between higher levels of empathy and the acceptance of online hate speech (Celuch et al., 2022; Wachs et al., 2022). Furthermore, adolescents who show understanding and emotional support towards peers targeted by hate speech are more likely to oppose it (Wachs et al., 2022). These findings are consistent with extensive research in related fields, which has consistently demonstrated that increased empathy correlates positively with defending victims of bullying (Álvarez-García et al., 2021; Gönültaş and Mulvey, 2023; Shen et al., 2023).

With the rise of social media and other digital platforms, individuals who actively express their opinions online may become more susceptible to hate speech. However, the influence of being a target of such hate speech on one’s attitude towards supporting regulatory measures is not extensively researched. This study seeks to explore how online engagement, such as posting content or uploading images, affects individuals’ support for regulations. It also aims to understand the influence of online activities on regulatory views, particularly for those who have experienced hate speech attacks. Based on these considerations, the study proposes the following research questions:

R3-1. Does online activity correlate with a higher level of agreement with hate speech regulations?

R3-2. Is online activity, when coupled with the experience of being victimized by hate speech, associated with a positive effect of supporting regulatory measures?

Prior research indicates that individuals who believe media content has a more pronounced effect on others than themselves are often inclined to endorse government media regulations (Gunther, 1995; Golan and Day, 2008). Sun et al. (2008), in their meta-analysis, found that exposure to undesirable media content, like online pornography and televised violence, tends to lead to support for restrictive and corrective actions. However, there is a notable research gap in understanding whether the belief in the efficacy of regulation, alongside the assessment of harmful content, impacts support for such regulation. Moreover, there is limited research on individuals’ perceptions of the effectiveness and their level of support for self-regulation by digital platforms, particularly in scenarios where government regulation is absent. This study hypothesizes that the perception of effectiveness in regulating online hate speech is a critical factor influencing support for such regulation. Hence, the following hypothesis regarding evaluation components is proposed:

*H2*. A positive evaluation of the effectiveness of methods to regulate online hate speech increases the likelihood of supporting the online regulation of hate speech.

## 3 Data and methods

### 3.1 Data

This study collected data through an online survey titled “A Survey of Korean Citizens’ Perception on Online Hate Speech,” conducted in February 2023 with the assistance from a polling firm M-Lab.^1^ This data was compiled from responses of volunteers participating in a survey conducted by M-Lab, using a respondent pool representative of the adult population in Korean society. The gender distribution among respondents was nearly even, with 50.9% male and 49.1% female participants. The respondents’ ages ranged from 18 to 69 years, with the following age distribution: 19.4% were 20 years old or younger, 17.4% in their 30s, 21.1% in their 40s, 22.7% in their 50s, and 19.4% in their 60s. In terms of educational background, 19.8% had completed high school or less, 68% had a university diploma, and 12.2% had a graduate degree or higher. Politically, the respondents identified as 1.9% progressive, 18.9% rather progressive, 56.6% moderate, 20.3% rather conservative, and 2.3% conservative. Regarding online activity, 16.6% were categorized as active, regularly posting content such as articles or photos online, while 83.4% were classified as nonactive, typically reading content uploaded by others or having a limited online presence (Table 1).

**Table 1:** Socio-demographics characteristics of the participants in the survey (*n* = 1,000).

Variable		*n*	%
Sex	Male	509	50.9
	Female	491	49.1
Age	20’s (including 18, 19 years old)	194	19.4
	30’s	174	17.4
	40’s	211	21.1
	50’s	227	22.7
	60’s	194	19.4
Level of education	High school graduate or less	198	19.8
	University	680	68.0
	Graduate school or higher	122	12.2
Political affiliation	Progressive	19	1.9
	Rather progressive	189	18.9
	Moderate	566	56.6
	Rather conservative	203	20.3
	Conservative	23	2.3
Online activity	Active (uploading contents)	166	16.6
	Non-active (not uploading contents)	834	83.4

### 3.2 Measures

#### 3.2.1 Online activity

The online activity (OA) variable reflects a user’s active behavior on the Internet. It is measured by whether they post articles or photos online (1 = “uploading online content,” 0 = “not creating any online content”).^2^

#### 3.2.2 Frequency of being a victim of online hate speech

Frequency of being a victim of online hate speech (Fq_Vic) was measured using nine items regarding the frequency of being attacked online. Respondents were asked if they had ever been targeted by online hate speech because of a specific identity among their many identities (0 = “no experience of attack” to 4 = “being attacked all the time”). Each identity originated from nine domains: gender, age, sexual orientation, place of origin, nationality/race, economic status, disability/illness, religion, and educational level. Each measurement question was analyzed as a single latent variable in the confirmatory factor analysis (CFA) model.

#### 3.2.3 Assessment on the severity of social harm caused by online hate speech

The severity of social harm caused by online hate speech (ASS_Harm) was assessed in two steps. For the first step, respondents were asked to indicate the negative impact of four types of online hate speech: “negative bias and stereotyping,” “expressions of contempt, harassment, and ridicule,” “hostile exclusion, discriminatory language,” and “threats, incitement to violence expression.” Each type of hate speech was indicated by five response items: (This type of online hate speech) (1) “reduces freedom of expression of social minorities,” (2) “generates and reinforces discrimination,” (3) “causes or exacerbates social conflict,” (4) “can lead people to violent behavior or crime,” or (5) “has no substantial effect.” Respondents were able to choose multiple statements that they thought were the result of online hate speech. However, if a respondent chose “(5) has no substantial effect,” the other statements could not be selected. Second, the number of negative responses was summed for each type of online hate speech. Consequently, each type of hate speech can have a negative social effect level on a scale of 0 to 4. Each score was used to create a latent variable in the CFA model (Table 2).

**Table 2:** Results of the measurement model.

	Number of items	Reliability		AVE
		α	CR	
1. Agreement on regulation	9	0.92	0.92	0.56
2. Assessment of social harm	4	0.92	0.92	0.73
3. Frequency of victimization	9	0.89	0.89	0.48
4. Assessment of regulation effectiveness	4	0.66	0.65	0.33

#### 3.2.4 Assessment on the effectiveness of ways to regulate online hate speech

The effectiveness of regulatory methods on online hate speech (Ass_RegEffect) was assessed using four items. These items are indicating effectiveness of the widely recognized regulatory methods on online hate speech^3^: “Mark the comments with a caution,” “Hide the comments so that users must click on it to see,” “Delete the comment,” and “Restrict activity of the commenter.” Each item was measured on a 5-point scale (1 = “no effect at all” to 5 = “very effective”). Finally, each item was used to create one latent variable in the CFA model (Table 2).

#### 3.2.5 Level of agreement on the need to regulate online hate speech

Level of agreement on the need to regulate online hate speech (Lv_RegAgr) was measured by nine items regarding targeted areas that online hate speech attacks: gender, age, sexual orientation, place of origin, nationality/race, economic status, disability/illness, religion, and education level. Respondents were asked to indicate what level of regulation was being requested for online hate speech for each of the nine domains (1: “no regulation is needed” to 5: “very strong regulation is requested”).

#### 3.2.6 Control variables

Sex, age, educational level, and political affiliation were also included in the analysis as control variables. The age variable was entered on a 6-point categorical scale (1 = 18–19 years old, 2 = 20s …. 6 = 60s). Education level was entered on a 3-point scale (1 = high school or less to 3 = graduate school or higher). Political affiliation was measured on a 5-point scale (1 = progressive to 5 = conservative).

### 3.3 Method

**Figure 1:**
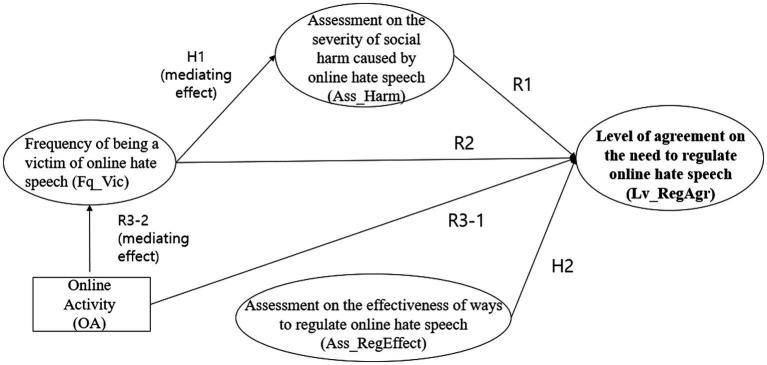
Theoretical path model of this study.

A structural equation model was used to test the theoretical model explaining the level of agreement with online regulations. It combined measurement and path models (Figure 1). The analysis was conducted using the “lavaan” package in R software (version 4.2.1).

## 4 Results

### 4.1 Evaluation of the measurement model

CFA was conducted on the measurement variables comprising the four latent variables for the measurement model. They are the frequency of being a victim of online hate speech (hereinafter, “Frequency of Victimization”), assessment of the severity of social harm caused by online hate speech (hereinafter, “Assessment of Social Harm”), assessment of the effectiveness of ways to regulate online hate speech (hereinafter, “Assessment of Regulation Effectiveness”), and level of agreement on the need to regulate online hate speech (hereinafter, “Agreement on Regulation”).

The three latent variables, except for Frequency of Victimization, were measured by the respondents’ attitudes toward various forms of online hate speech. The Frequency of Victimization was measured by the respondents’ experiences with different characteristics targeted by hate speech online (gender, age, race, disability, etc.).

The results of the CFA are presented in Table 2. The factor loadings of all variables were over 0.8, except for, “the Assessment of Regulation Effectiveness.” However, we can refer to the discussion that a factor loading value over 0.6 is sufficient in some cases (Bagozzi and Yi, 2012). CR values and AVE (Average Variance Extracted) values of all indexes except *Assessment of Regulation Effectiveness* were over 0.8 and 0.5 respectively, however, “Assessment of Regulation Effectiveness” could not meet the criteria. Given the theoretical goal of reflecting citizen evaluations of various regulatory options, we decided to retain the *Assessment of Regulation Effectiveness* variable in our model. Instead, we checked the model’s overall goodness-of-fit index and decided whether it would pass the test.

As shown in Table 3, the overall model fit index confirmed the goodness of fit of the CFA model (*χ*^2^ = 1240.272 (df = 401, *p* < 0.000), CFI = 0.936, TLI = 0.926, RMSEA = 0.046, GFI = 0.921, SRMR = 0.041). The correlations between the four latent variables and the online activity variable, which are the main variables in the analysis, are presented in Table 3.

**Table 3:** Correlation between key variables.

	1	2	3	4	5
1. Agreement on regulation	–				
2. Assessment of social harm	0.161***	–			
3. Frequency victimization	0.130***	0.078*	–		
4. Assessment of regulation effectiveness	0.283***	0.039	0.053	–	
5. Online activity	0.105***	0.076*	0.207***	0.029	–

### 4.2 Test of the structural model and hypothesis

**Figure 2:**
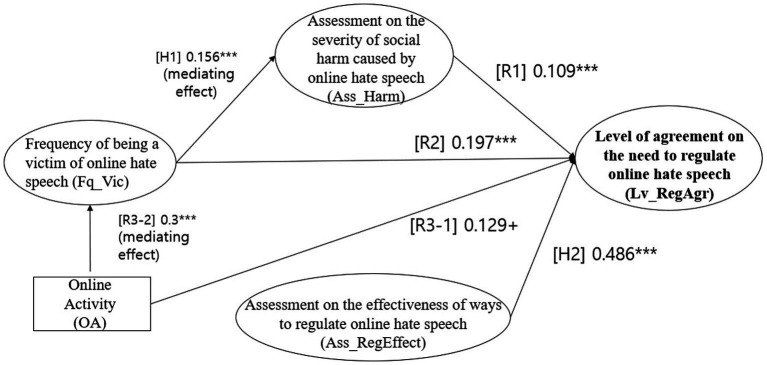
Coefficients of the path model.

After validating the structural equation path model (SEM), the model fit was found to be adequate. The goodness-of-fit of the structural equation model was *χ*^2^ = 1,240 (df = 401, *p* ≤ 0.001), CFI = 0.936, TLI = 0.926, RMSEA = 0.046, SRMR = 0.041. As shown in Figure 2, three of the main research questions and hypotheses of the study (R1, R2, and H2) were all found to be statistically significant at the 0.05 level of significance. However, R3-1 was found to be statistically significant at the 0.1 level of significance.

As shown in Table 4, the effect coefficients for each variable are as follows. The first set of variables to be examined are those that affect the level of agreement with the regulation of online hate speech. First, the greater the perceived social harm of online hate speech, the higher the level of agreement with regulation (*b* = 0.109, *p* < 0.001). Second, higher levels of victimization were associated with higher levels of agreement with the regulations (*b* = 0.197, *p* < 0.000). Third, those who are active online, writing posts or posting photos, were more likely to agree with the regulations than those who are not (*b* = 0.129, *p* < 0.055). Fourth, the more effective the online regulation is perceived to be, the stronger the agreement that regulation is needed (*b* = 0.486, *p* < 0.000).

**Table 4:** Results of the analysis.

Response var.	Independent var.	Estimate	SE	*P*	Hypothesis/research question results
Lv_RegAgr	Ass_RegEffect	0.486	0.085	0.000	Supported (H2)
	Ass_Harm	0.109	0.033	0.001	Sig. (R1)
	Fq_Vic	0.197	0.055	0.000	Sig. (R2)
	OA	0.129	0.067	0.055	Sig.(*p* < 0.1)(R3-1)
	Gender	−0.130	0.053	0.014	(Control var.)
	Age	−0.040	0.019	0.034	(Control var.)
	Education	−0.018	0.047	0.701	(Control var.)
	Political leaning	−0.129	0.036	0.000	(Control var.)
Ass_Harm	Fq_Vic	0.156	0.053	0.003	Supported (H1)
Fq_Vic	OA	0.300	0.056	0.000	Sig.(R3-2)
R-squared					
Lv_RegAgr	14.4%				
Ass_Harm	5.1%				
Fq_Vic	5.8%				

In addition, gender, age, and political affiliation significantly influenced the level of agreement with the regulation of online hate speech. Women (*b* = −0.130, *p* < 0.014), younger people (*b* = −0.040, *p* < 0.034), and people with more liberal political leanings (*b* = −0.129, *p* < 0.000) were more likely than men to agree with regulating online hate speech.

The path model also examined the impact of the frequency of victimization on the evaluation of its social harm. The results showed that the more severe the online hate speech attack, the higher the perceived social harm of online hate speech (*b* = 0.156, *p* < 0.003).

We also examine the impact of being active online on the victimization experience. Those who were active online, were a victim of online hate speech more frequently than those who were inactive (e.g., rarely using online media or just viewing other people’s posts) (*b* = 0.300, *p* < 0.000).

### 4.3 Assessment of the mediating effect

This study examined the direct and mediated effects of being a victim of hate speech and being active online. First, the respondents rated the negative effects of online hate speech as greater when they experienced attacks in online spaces because of their identity, which led to higher levels of agreement with online regulations. In other words, the victimization experience had a significant effect on attitudes towards regulating online hate speech, which was mediated by the evaluation of social harm [indirect effect of pathway (A): *b* = 0.017, *p* < 0.040].

Being active online also influences attitudes towards online regulation by affecting the likelihood of experiencing hate speech attacks. We found that engaging in creative activities such as writing posts or posting photos was associated with increased frequency of victimization experience. By mediating these effects, we found that active online content activity increases the level of agreement with online regulation. This pathway can be categorized into pathway (B), in which the activity affects online regulation by influencing the victimization experience, and pathway (C), in which the activity nourishes the victimization experience and finally affects online regulation by influencing the evaluation of social harm. Both pathways are significant at the 0.01 and 0.1 level of significance, respectively [indirect effects of pathway (B): *b* = 0.059, *p* < 0.003; indirect effects of pathway (C): *b* = 0.005, *p* < 0.059].

## 5 Discussion

### 5.1 The impact of being a victim of online hate speech

As online spaces develop and people spend more time online, the instances of being a victim of hate speech increases. This trend impacts people’s overall perceptions and attitudes toward online hate speech. Applying desensitization theory to online hate speech, it can be expected that heightened exposure to such content could lead to a perception of it as a customary element of online communication. Consistent exposure to hate speech, moreover, may influence individuals’ attitudes and beliefs towards certain groups. Over time, individuals might internalize and adopt the negative stereotypes and biases propagated by such speech, consequently influencing their perspectives and outlooks. Desensitization effect theory provides insight into how continuous exposure to harmful content affects individuals’ attitudes and beliefs. Past empirical studies focused on exposure to harmful content mainly in terms of consumption, rather than direct victimization experience. This study, however, delves into how being a victim of online hate speech, or the victimization experience, shapes individuals’ attitudes and beliefs.

Our findings indicate that victimization experience positively correlates with heightened awareness of the social harm of hate speech and increased support for its regulation (Table 5). We identified two mechanisms: direct support for regulation due to increased victimization, and an indirect pathway where victimization heightens awareness of social harm, leading to greater regulatory support. Those more victimized tend to perceive and evaluate the social harm of online hate speech more acutely, evident in stereotypes, ridicule, and intimidation. This research emphasizes the need to distinguish between types of media exposure in studies examining media’s influence. It is important to differentiate between exposure to fictional violence on TV and actual victimization in online environments. In the former, individuals are never direct victims, while in the latter, some individuals experience real victimization.

**Table 5:** Mediation analysis results (indirect effect).

Indirect path	Indirect effect	SE	*z*-value
[A] Fq_Vic → Ass_Harm → Lv_RegAgr	0.017*	0.008	2.053
[B] OA → Fq_Vic → Lv_RegAgr	0.059**	0.020	2.921
[C] OA → Fq_Vic → Ass_Harm → Lv_RegAgr	0.005+	0.003	1.891

This study findings also suggest that society’s negative perceptions of online hate speech are not merely derived from ethical and moral considerations but are a reality assessment that reflects the actual harm experienced by members of society. It is important to consider that regulatory attitudes towards online hate speech go beyond the rationale that “hate speech is harmful to society” and are linked to the experiences of those affected by it (Goodman, 2015; European Commission, 2017; Penney, 2017).

The identification of both direct and indirect pathways leading to greater support for regulation indicates a complex interplay between exposure, understanding of consequences, and advocacy for regulation, highlighting the multifaceted nature of how exposure influences attitudes and actions. Moreover, the observation that those frequently targeted by online hate speech are more likely to perceive its severe social impacts underscores the psychological and emotional toll on victims. This highlights the potential long-lasting effects on victims’ well-being and self-perception, as well as the wider societal implications of hate speech.

The study’s findings reinforce the necessity for comprehensive strategies to tackle online hate speech. Efforts to increase awareness of its detrimental effects, enhance media literacy, and establish legal frameworks for regulation are all vital in creating a safer and more respectful online space.

### 5.2 Implications of being active online

Traditionally, online spaces have been portrayed as places of free expression and interconnection. This orientation is related to the fact that “freedom of expression” is often the most important point of contention when it comes to regulating online spaces. The opposition to regulating online hate speech, or inaction in regulating online hate speech, is based on the idea that regulation will infringe on freedom of expression online. The idea is that curtailing freedom of expression leads to a decrease in free speech and online expression.

From this perspective, users who actively upload online content, such as writing posts or uploading photos, can be expected to have a negative attitude towards the regulation of online spaces (Ham and Lee, 2020). This is because they are more likely to perceive hate speech regulation online as restricting their ability to be active online. However, another way to look at this is that the more active one is online, the more incentives one has to ensure that the space remains in a healthy environment. The more active they are online, the more stakes they have in the soundness of the online space.

We found that being active online is associated with higher levels of agreement with online regulations, and this effect occurs directly and indirectly, which can be interpreted as follows: first, active online presence such as uploading contents may be associated with a higher perceived need for regulation because of concerns about healthy online spaces, as discussed earlier. It can be argued that uploading content online also leads to a proactive attitude towards operating and managing online spaces. This implies that people who are directly involved in online interactions and communities may recognize the potential negative aspects of online platforms and advocate for measures to address them. Second, we found an indirect effect of types of online activity on regulatory attitudes, mediated by the experience of being attacked by online hate speech. The more people active online, such as posting content, the possibility they could be victimized by online hate speech increases. This suggests that the more active a person is in an online space, the more likely they are to be a victim of hate speech. This implies that being active online increases the chance of victimization experience, which increases both the perceived social harm of hate speech and the level of agreement with regulations.

### 5.3 Toward a social consensus on regulating online hate speech

Amid the growing necessity to regulate online hate speech, there’s a delicate balance to maintain with users’ freedom of expression, which has resulted in less active enforcement of restrictions. Online hate speech’s detrimental impacts have escalated into a worldwide concern. Consequently, garnering a stronger consensus among users may be imperative for regulating online hate speech compared to other areas. This study aims to shed light on the factors that shape people’s support for such regulations, thereby aiding in building a robust consensus.

We focused on the influence of perceived regulatory effectiveness on attitudes toward online hate speech regulation. Regulatory theory posits that individuals’ attitudes towards regulation are influenced not only by the perceived need for such regulation but also by its expected effectiveness. According to research (Nielsen and Mathiesen, 2003), even when there is a recognized need for regulation, people may be hesitant or opposed to it if they anticipate its ineffectiveness. Our analysis concurs, revealing that the extent of support for regulating online hate speech is significantly shaped by expectations of its efficacy. This underscores the importance of effective regulation in building citizen consensus.

This finding has critical implications for policymakers and advocates working towards regulating online hate speech. It highlights the necessity of effectively communicating the efficacy of proposed regulations. Transparent communication about how these regulations will tackle the issue and the metrics for evaluating their impact can significantly sway public opinion and support (Braman and Kahan, 2003; Gollust et al., 2013). Clearly articulating the functionality and benefits of these regulations can play a pivotal role in enhancing public backing and ensuring successful implementation.

## 6 Conclusion and limitation

This research delves into the impact of online hate speech on users, suggesting that as hate speech proliferates, there is a tendency for increased social instability. It highlights that individuals who are frequently targeted by online hate speech often have a heightened awareness of its social harms. The study reveals that citizens’ support for online regulation is influenced by two key social factors: the impact of online hate speech and the perceived effectiveness of regulatory measures. The key determinants for supporting online hate speech regulation include individual online experiences, assessments of the social harm caused by online hate speech, and beliefs about the effectiveness of regulation. These empirical findings offer significant insights for fostering a consensus among citizens on the regulation of online hate speech.

Despite its valuable contributions, it is crucial to recognize certain limitations of this study, particularly concerning its applicability beyond the Korean context. First, the focus on Korean citizens might restrict the generalizability of the findings, as cultural differences, linguistic nuances, and varied online behaviors across different societies could alter the applicability of these insights. Cultural norms at the national level, such as collectivism versus individualism, can greatly influence attitudes towards the regulation of online hate speech. Additionally, a country’s historical and political backdrop can shape its citizens’ perspectives on regulation. For example, societies with a history of authoritarianism might be more cautious about government interventions. Although the patterns and effects of online hate speech may have universal elements, and the perceived harm by victims could be higher regardless of geographical location, the attitudes towards regulation are likely to be heavily influenced by cultural, political, social, and historical contexts. Therefore, while this study offers valuable perspectives, caution should be exercised in generalizing its findings, acknowledging the influence of these varying contextual factors.

Secondly, this study’s reliance on self-reported data introduces potential biases and inaccuracies due to the subjective nature of the responses. The possibility of self-selection bias, where participants more sensitive to online hate speech might disproportionately represent the sample, could also skew the results. Moreover, as is the case with any empirical research, this study’s conclusions are constrained by the limitations inherent in the research methodologies used. Despite efforts to control for confounding variables and conduct rigorous statistical analysis, the dynamic and complex nature of the online environment, coupled with the evolving characteristics of hate speech, might lead to unconsidered variables that could impact the study’s findings. Additionally, the survey measures employed were not specific to any particular digital platform. Since the responses were based on individuals’ general online experiences, the findings can be generalized to the broader online experiences of Korean society. However, this approach has limitations when it comes to applying the results to specific platforms where hate speech is more prevalent. The unique dynamics and user interactions of each platform may significantly affect the experience and perception of hate speech, which this study may not fully capture.

Lastly, the scope of this study was primarily exploratory, given the relatively nascent state of research on the topic of citizens’ attitudes towards the regulation of online hate speech. While we have developed a comprehensive model by examining the interplay between various factors such as citizens’ experiences, perceptions, and evaluations of online hate speech, the theoretical underpinnings that explain the connections between these variables still require further development and reinforcement. Additionally, the data utilized in this study is cross-sectional in nature, which inherently limits the ability to establish causality between the variables. This limitation underscores the need for ongoing and more extensive research in this area to draw stronger causal inferences.

Despite these constraints, our study serves as an important foundational work for future research endeavors aimed at deepening the understanding of the factors that shape people’s attitudes towards the regulation of online hate speech. Recognizing the limitations outlined above, we advise a cautious and nuanced interpretation and application of our findings. This study will stimulate further discussion and research in the ever-evolving field of online hate speech and its regulation, ultimately leading to more informed and effective policymaking.

## Data availability statement

The raw data supporting the conclusions of this article will be made available by the authors, without undue reservation.

## Ethics statement

The study was approved by the Institutional Review Board of Seoul National University. The study was conducted in accordance with local legislation and institutional requirements. Informed consent to participate in this study was secured by the polling organization that conducted the research survey.

## Author contributions

AP: Project administration, Writing – original draft. MK: Conceptualization, Writing – review & editing. E-SK: Formal analysis, Writing – review & editing.

## Footnotes

^1^The data collection was carried out in line with Kakao’s self-regulatory measures pertaining to online hate speech, and Kakao supported the survey’s execution.

^2^Related studies often measure online activity on multiple dimensions and then apply dimension reduction techniques. However, in this study, we used a single item because we intended to measure the direct effect of the activity itself, which is when people produce content in online spaces. When measuring a specific online activity like this, there are previous studies that have used a single item to measure behavior (Ko, 2018; Kim et al., 2020).

^3^Each of the four regulatory methods is a way for platform companies to be regulated by online platforms, which is widely recognized in Korea.
